# Kisspeptin Influences the Reproductive Axis and Circulating Levels of microRNAs in Senegalese Sole

**DOI:** 10.3390/ijms21239051

**Published:** 2020-11-28

**Authors:** Catarina C. V. Oliveira, Elvira Fatsini, Ignacio Fernández, Catarina Anjos, François Chauvigné, Joan Cerdà, Robin Mjelle, Jorge M. O. Fernandes, Elsa Cabrita

**Affiliations:** 1 Center of Marine Sciences-CCMAR, University of Algarve, 8005-139 Faro, Portugal; effernandez@ualg.pt (E.F.); cmanjos@ualg.pt (C.A.); 2 Aquaculture Research Center, Agrarian Technological Institute of Castile and Leon, Ctra. Arévalo, s/n, 40196 Segovia, Spain; FerMonIg@itacyl.es; 3 IRTA-Institute of Biotechnology and Biomedicine (IBB), Universitat Autònoma de Barcelona, 08193 Barcelona, Spain; francois.chauvigne@irta.cat (F.C.); joan.cerda@irta.cat (J.C.); 4 Faculty of Bioscience and Aquaculture, Nord University, 8049 Bodø, Norway; robin.mjelle@ntnu.no (R.M.); jorge.m.fernandes@nord.no (J.M.O.F.)

**Keywords:** *Solea senegalensis*, G1 breeders, hormonal treatment, KISS2, gonadotropins, sex steroids

## Abstract

Kisspeptin regulates puberty and reproduction onset, acting upstream of the brain–pituitary–gonad (HPG) axis. This study aimed to test a kisspeptin-based hormonal therapy on cultured Senegalese sole (G1) breeders, known to have reproductive dysfunctions. A single intramuscular injection of KISS2-10 decapeptide (250 µg/kg) was tested in females and males during the reproductive season, and gonad maturation, sperm motility, plasma levels of gonadotropins (Fsh and Lh) and sex steroids (11-ketotestosterone, testosterone and estradiol), as well as changes in small non-coding RNAs (sncRNAs) in plasma, were investigated. Fsh, Lh, and testosterone levels increased after kisspeptin injection in both sexes, while sperm analysis did not show differences between groups. Let7e, miR-199a-3p and miR-100-5p were differentially expressed in females, while miR-1-3p miRNA was up-regulated in kisspeptin-treated males. In silico prediction of mRNAs targeted by miRNAs revealed that kisspeptin treatment might affect paracellular transporters, regulate structural and functional polarity of cells, neural networks and intracellular trafficking in Senegalese sole females; also, DNA methylation and sphingolipid metabolism might be altered in kisspeptin-treated males. Results demonstrated that kisspeptin stimulated gonadotropin and testosterone secretion in both sexes and induced an unanticipated alteration of plasma miRNAs, opening new research venues to understand how this neuropeptide impacts in fish HPG axis.

## 1. Introduction

The kisspeptin system is widely known to control puberty and to be involved in the onset of reproduction in mammals, acting centrally via the kisspeptin receptor and stimulating the secretion of gonadotropin-releasing hormone (GnRH) [1,2]. In humans, kisspeptin treatments have been proven to be a very promising therapeutic in the treatment of fertility disorders, as it stimulates the release of gonadotropins [1]. In subfertile women, it has been observed to induce oocyte maturation [3] and in men, intravenous administration of two different kisspeptin isoforms stimulated the levels of pituitary gonadotropins luteinizing (Lh) and follicle-stimulating (Fsh) hormones in serum [4]. 

In fish, as in mammals, the kisspeptin system seems to play a major role in the regulation of the gonadotropic axis, especially in the timing of puberty and control of gonadotropin secretion [5,6,7]. In most teleost fish, the kisspeptin system is composed of two ligands, KISS1 and KISS2, and two receptors, KISS2r and KISS3r (reviewed in Somoza*,* et al. [8]). Although KISS2 appears to have a predominant role in the control of reproduction [9,10], both kisspeptin peptides have been demonstrated to stimulate gonadotropin synthesis and secretion in different fish species, accelerating puberty in juveniles or gametogenesis in adults [9]. However, their actions do not appear to be mediated by GnRH neurons as in mammalian models (reviewed in Somoza*,* et al. [8]). Acting upstream of the hypothalamus–pituitary–gonad (HPG) axis, this hormonal treatment could represent a valuable tool to optimize current breeding protocols in commercial cultured fish.

The Senegalese sole, *Solea senegalensis*, is an emerging and promising species for European aquaculture [11]. However, the lack of fertilization of spawned eggs from broodstock bred and reared in captivity (first generation, G1) is one of the major constraints [12,13]. Such reproductive failure has been attributed in part to males, based on the fact that G1 males lack the courtship behavior observed in wild breeders [14]. However, lower levels of Lh [15] and of sperm volume and quality [16], with respect to wild individuals, are also factors involved. Some advances have been achieved to temporarily solve these problems, namely the development of artificial fertilization [17,18] or cryopreservation protocols [19], as well as the improvement of the reproductive status through nutritional approaches [20]. Hormonal induction in G1 sole has shown partially successful results; in females with gonadotropin-releasing hormone agonists (GnRHa) slow-release implants [18,21] and in males with human chorionic gonadotropin (hCG) [22] or homologous recombinant gonadotropins [23,24] injections. Hormonal therapies could provide a solution for the reproductive dysfunctions, but improvement is still insufficient and further research is still needed. 

In Senegalese sole, the expression of only *kiss2* gene (*Sskiss2*) has been identified so far, although two *sskiss2* mRNA splice forms are detected: *Sskiss2_v1*, which produces a functional protein, and *Sskiss2_v2*, which encodes for a truncated, non-functional protein [25]. In this species, not only temporal and spatial, but also sex-specific differences in transcript levels were found. In males, *kiss2* and its receptor were more expressed in the brain towards the end of winter, just before the spawning season, coinciding with the highest levels of *fshb* and *lhb* subunit mRNAs in the pituitary, and of plasma levels of testosterone (T) and 11-ketotestosterone (11-KT) [26]. 

Over the last years, several studies have reported the key role of non-coding RNAs (ncRNAs) in the regulation of gene expression and translation in multicellular organisms [27]. MicroRNAs (miRNAs) are small single-stranded ncRNAs (21–25 nucleotides (nt), reviewed in [28]) playing crucial roles in response to environmental changes, specific treatment and/or disease [29,30] and are involved in different fundamental biological processes, including reproduction [31,32]. Many miRNAs have been found in the blood plasma of various organisms, including mammals and fish [20,33]. Moreover, the blood plasma miRNAs might be used as timely, sensitive biomarkers for several biological processes [34]. In fish, differentially expressed (DE) miRNAs related to sex were observed in serum in the tongue sole (*Cynoglossus semileavis*) [35]. In common carp (*Cyprinus carpio*), several circulating miRNAs were expressed after exposure to a potent herbicide [36]. Specifically, in case of the Senegalese sole, there is evidence that vitamin K supplementation improves sperm quality and an additional complex tissue crosstalk along the HPG axis might take place through some small non-coding RNAs (sncRNAs) in blood plasma [20]. Currently, to our knowledge, there is no information on how the treatment with kisspeptin may affect sole reproduction and which levels of the HPG axis it would affect, including the regulation of gene expression through sncRNAs. Hence, we performed an integrative analysis, including gonadotropins and sex steroids plasma concentrations, gonadal development, sperm quality, and circulating miRNAs in blood plasma, after a kisspeptin treatment.

## 2. Results

### 2.1. Kisspeptin Treatment Affects the Reproductive axis

**Figure 1:**
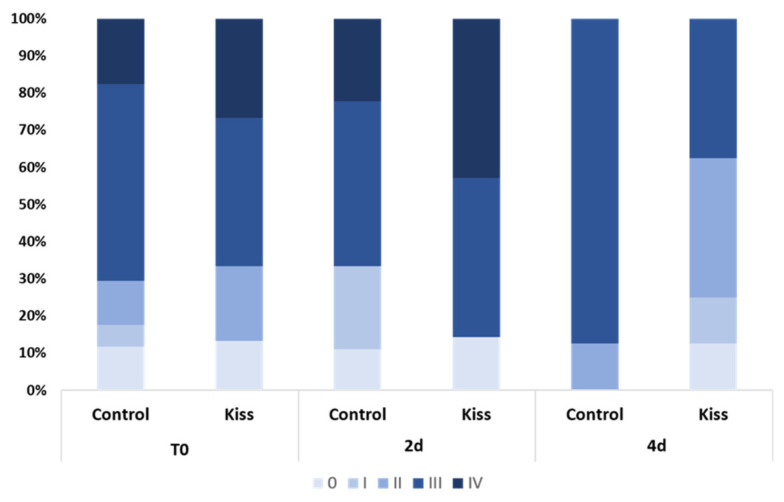
Percentage of gonad maturation stages (0 to IV) in Senegalese sole females treated with kisspeptin hormone (*n* = 15) and untreated controls (*n* = 17) before (T0) and after (2 d: 2-days, 4 d: 4-days) kisspeptin injection.

During the experimental period, several alterations on the gonad development, gonadotropins and sex steroids plasma levels, as well as on the expression levels of blood plasma miRNAs were observed in both genders. At the beginning of the experiment (T0), both female groups (Ccontrol and kisspeptin treatments) showed a similar percentage of maturation stages with the presence of more females in stages III and IV (Figure 1). Nevertheless, the group treated with kisspeptin seemed to present higher percentage of females in stage IV than control group at 2 days post-treatment (2 d). However, at 4 days post-treatment (4 d) no females in stage IV were observed in both groups.

**Figure 2:**
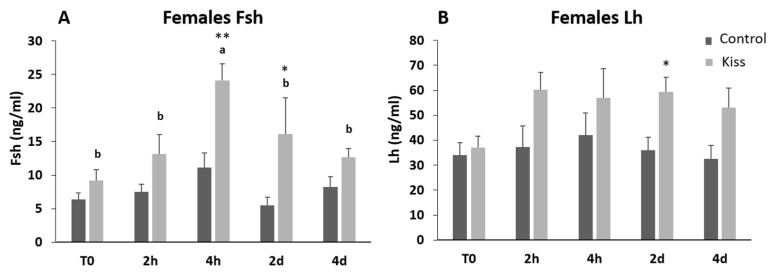
Gonadotropin plasma levels in ng/mL (mean ± standard error of the means, SEM) from Senegalese sole females treated with kisspeptin hormone (*n* = 15) and controls (*n* = 17). (**A**) Fsh and (**B**) Lh levels before (T0) and after (2 h: 2-h; 4 h: 4-h; 2 d: 2-days; 4 d: 4-days) the kisspeptin injection. The asterisk denotes significant differences between experimental groups at each sampling point (Student’s *t*-test; * *p* < 0.05; ** *p* < 0.002); different letters indicate significant differences among sampling points within each experimental group (one-way ANOVA, Dunnett’s test, *p* < 0.05, T0 considered as control point).

**Figure 3:**
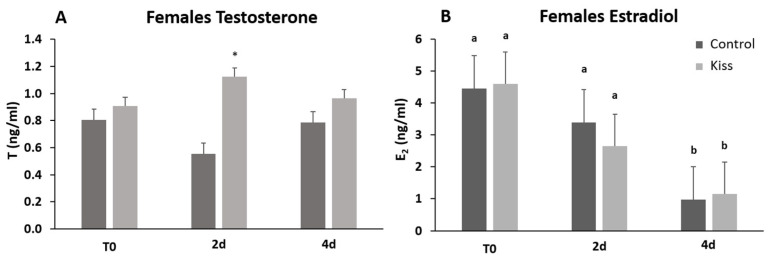
Sexual steroids plasma levels in ng/mL (mean ± SEM) from Senegalese sole females treated with kisspeptin hormone (*n* = 15) and control (*n* = 17). (**A**) Testosterone and (**B**) Estradiol levels before (T0) and after (2 h: 2-h; 4 h: 4-h; 2 d: 2-days; 4 d: 4-days) the kisspeptin injection. The asterisk denotes significant differences between experimental groups at each sampling point (Student’s *t*-test, *p* < 0.05); different letters indicate significant differences among sampling points within each experimental group (one-way ANOVA, Dunnett’s test, *p* < 0.05, T0 considered as control point).

Regarding Fsh plasma levels, they were significantly increased from T0 (9.22 ± 1.62 ng/mL), reaching a peak at 4 h post-injection (4 h) (24.11 ± 2.53 ng/mL, *p* = 0.0435, Figure 2A) in females treated with kisspeptin. This variation was not observed in the control group, and significantly lower levels of Fsh in plasma was noted at 4 h post-injection (11.10 ± 2.18 ng/mL) in comparison with kisspeptin treated females (*p* = 0.002, Figure 2A). Afterwards, Fsh plasma levels decreased, still being significantly higher in kisspeptin-treated females (16.16 ± 5.37 ng/mL) than in control group (5.47 ± 1.25 ng/mL, *p* = 0.03) at 2 d, but not at 4 d (Figure 2A). Concerning Lh plasma levels, although values in the kisspeptin group tend to increase from T0 (37.10 ± 4.47 ng/mL: Figure 2B) to 2 h (60.33 ± 6.76 ng/mL), no statistical differences were found along the sampling points. Nevertheless, at 2 d post-treatment, values in the kisspeptin group were significantly higher than in control group females (36.03 ± 5.13 ng/mL: Figure 2B, *p* = 0.014). The levels of T in females exhibited the same profile of Lh, being only significantly higher in kisspeptin-treated females (1.12 ± 0.27 ng/mL) than in control females (0.55 ± 0.09 ng/mL) at 2 d (Figure 3A, *p* = 0.03). The levels of estradiol (E_2_) decreased in both female groups from T0 to 4 d (*p* = 0.0127) and no differences were found between experimental groups at any sampling time (Figure 3B).

**Figure 4:**
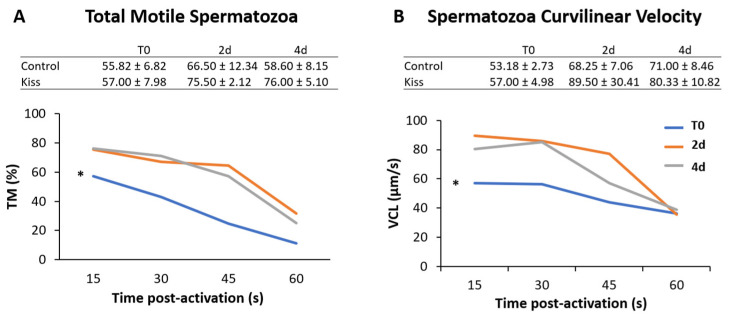
Sperm motility of Senegalese sole males treated with kisspeptin hormone (*n* = 17) and control (untreated) (*n* = 13). (Graphic) (**A**) Percentage of motile cells, total motile spermatozoa (TM) and (**B**) Curvilinear velocity, VCL in µm s-1 for 15, 30, 45 and 60 s post-activation, from treated males before (T0) and after (2 d: 2-days; 4 d: 4-days) the kisspeptin injection. (Table) (**A**) TM and (**B**) curvilinear velocity (VCL) for 15 s post-activation (mean ± SEM) from treated and control males at the same sampling points. The asterisk (*) denotes significant differences between sampling points (GLM, *p* < 0.05).

**Figure 5:**
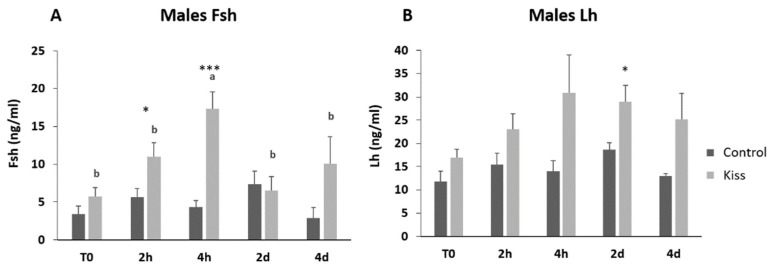
Gonadotropins plasma levels in ng/mL (mean ± SEM) from Senegalese sole males treated with kisspeptin hormone (*n* = 17) and control (*n* = 13). (**A**) Fsh and (**B**) Lh levels before (T0) and after (2 h: 2-h; 4 h: 4-h; 2 d: 2-days; 4 d: 4-days) the kisspeptin injection. The asterisk denotes significant differences between experimental groups at each sampling point (Student’s *t*-test; * *p* < 0.05; *** *p* < 0.001); different letters indicate significant differences among sampling points within each experimental group (one-way ANOVA, Dunnett’s test, *p* < 0.05, T0 considered as control point).

**Figure 6:**
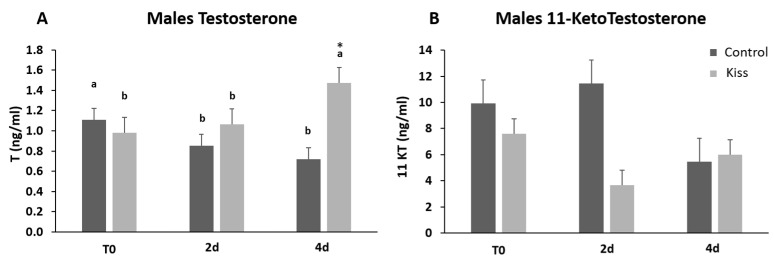
Sexual steroids plasma levels in ng/mL (mean ± SEM) from Senegalese sole males treated with kisspeptin hormone (*n* = 17) and control (*n* = 13). (**A**) Testosterone and (**B**) 11-Ketotestosterone levels before (T0) and after (2 h: 2-h; 4 h: 4-h; 2 d: 2-days; 4 d: 4-days) the kisspeptin injection. The asterisk (*) denotes significant differences between experimental groups at each sampling point (Student’s *t*-test, *p* < 0.05); different letters indicate significant differences among sampling points within each experimental group (one-way ANOVA, Dunnett’s test, *p* < 0.05, T0 considered as control point).

Regarding the effect on males, total motile spermatozoa (TM) and curvilinear velocity (VCL) were evaluated to infer the sperm quality in both groups. TM significantly improved in both male groups (control and kisspeptin) from T0 to 2 d and 4 d (Figure 4A, *p* = 0.001). However, no differences were noticed between control and kisspeptin groups. In the case of VCL, a similar pattern was observed. The velocity significantly increased in both male groups from T0 to 2 d and 4 d (Figure 4B, *p* = 0.002), but no significant differences were observed between experimental groups. Regarding cell viability, no differences were also observed between groups (Table 1). As to Fsh plasma levels, these levels significantly increased from T0 (5.71 ± 1.18 ng/mL, Figure 5A) to 4 h (17.34 ± 2.20 ng/mL) but decreased afterwards in males treated with kisspeptin. Values were significantly higher in comparison with the control group at 2 (5.63 ± 1.17 ng/mL, *p* = 0.039) and 4 h (4.36 ± 0.86 ng/mL, *p* = 0.0003, Figure 5A). The Lh plasma levels tended to gradually increase from T0 in males treated with kisspeptin, but only being significantly different at 2 d post-injection (28.94 ± 3.53 ng/mL) compared with the control group (18.68 ± 1.43 ng/mL, *p* = 0.028, Figure 5B). The levels of T in plasma of males treated with kisspeptin significantly increased from T0 (0.98 ± 0.14 ng/mL) to 4 d (1.47 ± 0.17 ng/mL, Figure 6A). In contrast, these levels gradually decreased in control males, being significantly different from T0 (1.11 ± 0.10 ng/mL) to 2 d (*p* = 0.0320). Besides, higher levels were found when compared kisspeptin-treated males (0.72 ± 0.04 ng/mL, *p* = 0.0024, Figure 6A) with control group at 4 d post-injection. No differences were observed in the 11-KT plasma levels within the same group or between groups at any sampling point (Figure 6B). 

### 2.2. Circulating sncRNA Profiles in Plasma

**Figure 7:**
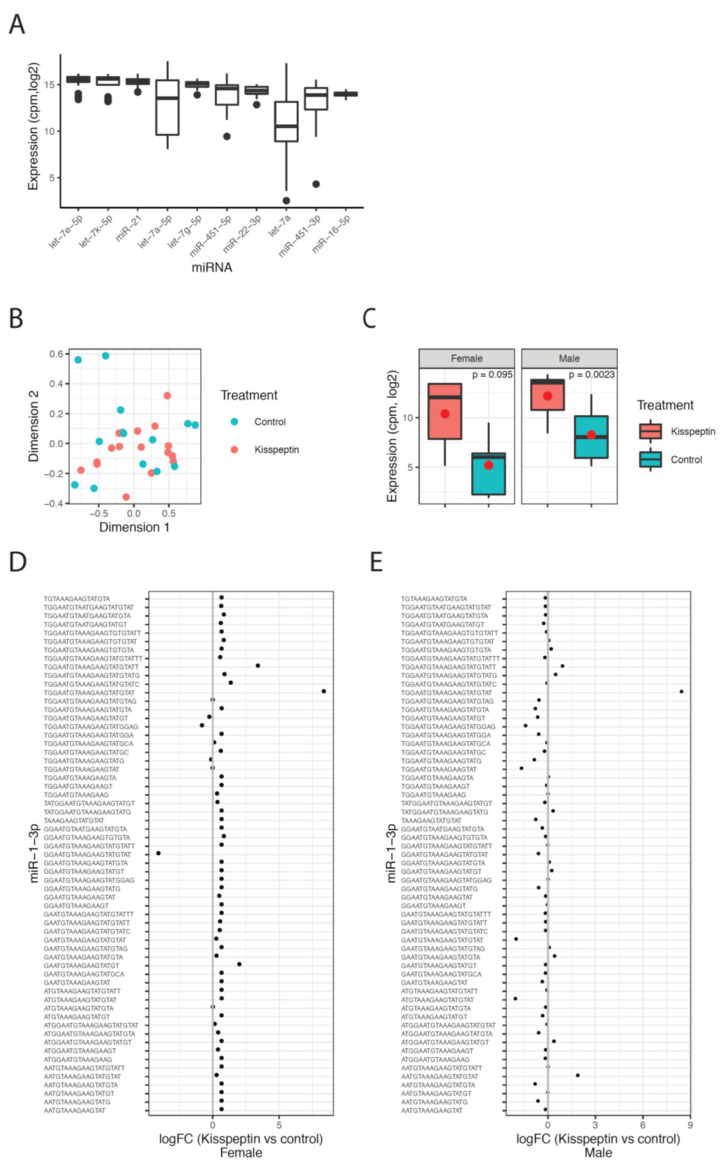
MicroRNA sequencing results. (**A**) Levels of the top 10 highest expressed miRNAs shown as the normalized expression (cpm, log2) for each miRNA across all samples. (**B**) Multidimensional scaling plot (MDS) of the miRNA expression data colored by treatment, red color for animals treated with kisspeptin and blue color for untreated fish. (**C**) Expression of miR-1-3p in kisspeptin and control treatments for female and male samples, respectively. The mean of each group is shown as a red circle. The *p*-values are the Benjamini–Hochberg adjusted *p*-values as calculated by *limma* in R. (**D**) Fold change values (log2) for all detected isomiRs of miR-1-3p between kisspeptin and control treatment in females. (**E**) Similar as in D for male samples.

The sequencing results revealed an enrichment of RNA fragments around 21–22 nt, indicating a high abundance of miRNA reads in the sequenced libraries (Supplementary Figure S1A). The reads of the hairpin sequence were mapped to miRNAs from all species in miRbase and the miRNA with the highest expression across all miRbase-species was defined as the correct miRNA. Using this approach, 1467 uniquely expressed miRNAs were identified in Senegalese sole. Let-7e-5p was the highest expressed miRNA, along with other members of the let-7 family (Figure 7A). 

Other ncRNAs were identified by first mapping the reads to the genome of smooth tongue sole and zebrafish (*Danio rerio*) followed by annotation of the aligned reads to the RNACentral database of ncRNAs, using the zebrafish gff-file. The most abundant ncRNAs belonged to the classes of ribosomal RNAs (rRNAs), transfer RNAs (tRNAs), and long-noncoding RNAs (lncRNAs) (Supplementary Figure S1). 

The effect of kisspeptin on the miRNAome was investigated by comparing the expression levels between the two experimental (kisspeptin and control) groups for males and females. A multidimensional scaling (MDS) plot revealed no clustering of the sequenced samples with respect to treatment, indicating that the absence of global changes in miRNA expression 2 d post-treatment in males and females (Figure 7B). Samples from males at 4 d post-treatment were not included in this analysis due to the no change among the experimental period. The differential expression analysis revealed one miRNA (miR-1-3p) that was up-regulated in males treated with kisspeptin compared to control males, and highly expressed but not significantly different in females (Figure 7C). Further, this difference was due to one sequence variant (isomiR) of miR-1-3p (Figure 7D,E). In total, 70 different isomiRs for miR-1-3p was detected, of which one isomiR was consistently up-regulated in both males and females (Figure 7D,E). Most of the other isomiRs for miR-1-3p showed minor changes between the treatment groups, indicating that this particular isomiR is responsible for the changes of miR-1-3p upon kisspeptin treatment. In females, three DE isomiRs (let-7e, miR-199a, miR-100) were found between kisspeptin-injected and control Senegalese sole females (Table 2). While let-7e and miR-199a were up-regulated (6.3- and 8.0-fold change, respectively) in females treated with kisspeptin, miR-100 was down-regulated (−9.3-fold change).

### 2.3. Bioinformatic Prediction of mRNAs Targets by Differentially Expressed (DE) miRNAs

**Figure 8:**
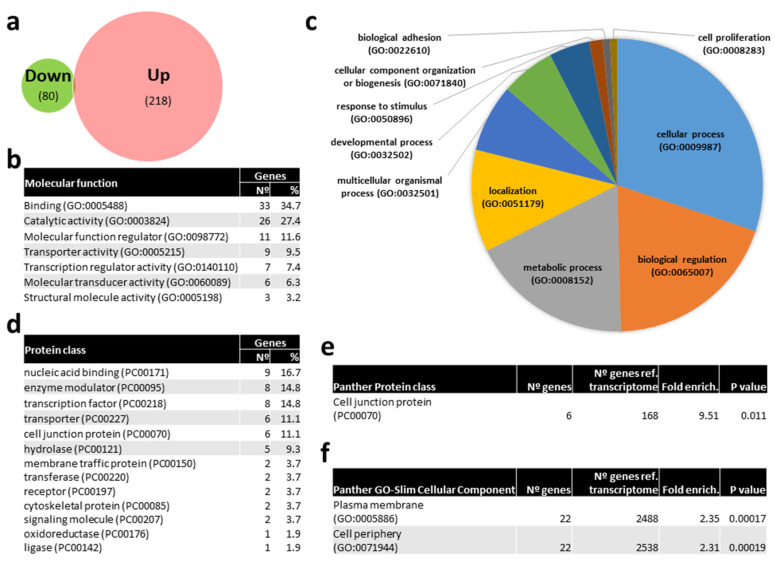
Venn diagram of predicted mRNA targets of miRNAs differentially expressed (DE) with kisspeptin treatment in blood plasma of Senegalese sole females and gene ontology (GO) analysis. (**a**) Venn diagram with the number of predicted mRNAs targeted by DE miRNAs. (**b**) List of GO molecular functions represented by predicted mRNAs targeted by DE miRNAs. (**c**) Pie chart of GO biological process of predicted mRNAs targeted by DE miRNAs. (**d**) List of GO Protein classes represented by predicted mRNAs targeted by DE miRNAs. (**f**) List of overrepresented Panther Protein classes (**e**) and GO-Slim Cellular Component of predicted mRNAs targeted by DE miRNAs showing the number of genes, the fold enrichment and the *p* value.

In order to get insights on the particular molecular pathways altered by kisspeptin, potential mRNAs targets of DE miRNAs were identified *in silico*. Only 4 mRNAs were predicted to be targeted by miR-1-3p, namely *DNA methyltransferase 3ab* (*dnmt3ab*), s*erine palmitoyl transferase, long chain base subunit 1* (*sptlc1*), *PQ loop repeat containing 3* (*pqlc3*), and *tropomyosin alpha-4 chain a* (*tpm4a*). In contrast, 218 and 80 mRNA transcripts (Figure 8A, Supplementary Table S1) were predicted to be targeted by up- and down-regulated miRNAs in kisspeptin-injected females, respectively. A gene ontology (GO) analysis of the corresponding 240 well annotated mRNAs, showed that binding (34.7%), catalytic activity (27.4%), molecular function regulator (11.6%), and transporter activity (9.5%) were the most abundant molecular functions among the 7 represented (Figure 8B). Among the 10 biological processes where the identified targets were included, cellular process (30.1%), biological regulation (19.5%), metabolic process (18.0%) and localization (11.3%) were the most represented (Figure 8C). mRNAs targets were clustered in 13 distinct protein classes, being nucleic acid binding (16.7%), enzyme modulator (14.8%), transcription factor (14.8%), transporter (11.1%), and cell junction protein (11.1%) the most abundantly represented (Figure 8C). Interestingly, the gonadotropin releasing hormone receptor pathway was found among the 36 pathways represented (Supplementary Table S2), including *MAP kinase kinase 4 b* (*map2k4b*) and *tyrosine-protein kinase receptor* (*insra*) genes. Furthermore, cell junction protein (9.51-fold enrichment, Figure 8E) class, and plasma membrane and cell periphery (2.35- and 2.31-fold enrichment, respectively, Figure 8F) GO-Slim cellular components were significantly overrepresented in females treated with kisspeptin. Among cell junction proteins, *tight junction protein ZO-3* (*tjp3*), *ocludin a* (*oclna*), and *gap junction protein* (*cx28.9*) were identified. Sortings, such as *sorting nexin-1* (*snx1a*) and *family member 27 a* (*snx27a*), *trafficking protein kinesin binding 1* (*trak1a9*), *Dopey family member 2* (*dopey2*), *erlin-1* (*erlin1*), transporters *slc6a22.1* and *slc17a7a*, *tyrosine-protein kinase receptor* (*insra*), *phosphatidylinositol-4,5-bisphosphate 3-kinase, catalytic subunit beta* (*pik3cb*), and *myosin XVI* (*myo16*) were amongst the overrepresented cellular components.

## 3. Discussion

In the present study, we demonstrated that a single dose treatment with kisspeptin affected the HPG axis at different levels, indicating the promising potential of this treatment to solve Senegalese sole reproductive dysfunctions. In addition, this is the first study reporting an effect of hormonal treatment on the levels of circulating miRNAs in fish, widening the previously reported identification of miRNAs as potential biomarkers in fish physiology, particularly for nutritional and reproductive status [20], and providing new clues on the mechanisms by which kisspeptin might impact the HPG axis in teleosts.

The KISS2-10 treatment administrated to Senegalese sole females induced a positive response on gonadotropin levels. Fsh elevation was sufficient to elicit a response from T at 2 d, but not to induce E2 production. The increase in Lh levels seemed to induce oocyte maturation as well, since it was accompanied by an increase in the number of females in stage IV of gonadal maturation. In males, the single kisspeptin dose elicited a similar response. In this case, Fsh was significantly elevated at 2 and 4 h after administration and Lh was higher at 2 d post-treatment. The Fsh elevation influenced T, but this time a bit later, 4 days after treatment. The levels of 11-KT, though, were not altered by the treatment. Sperm motility parameters (TM and VCL) increased at 2 and 4 days after the treatment with kisspeptin, with a minor rise also observed in the control group, suggesting that the surge induced by the treatment could have an adjuvant like natural oscillations in water temperature or sperm renovation after first collection, inducing the production of fresh and high-quality sperm. This is likely related to the type of spermatogenesis in this species, semi-cystic and asynchronous, which happens gradually in successive batches [37]. Probably, multiple injections, higher doses and/or additional signals might be needed to fully trigger the reproductive axis, increasing sperm production and its quality. Consequently, also the reproductive courtship display in this species could be improved.

Actually, in two species of the genus *Morone*, multiple injections of kisspeptins increased spermatozoa production in juveniles and elicited gonadal development in sexually mature fish [38]. Although this hormonal therapy is far more advanced in humans [1,4], previous studies in fish have also reported positive effects on the HPG axis. In European seabass (*Dicentrarchus labrax*) a single intramuscular injection of KISS2-10 decapeptide increased the production of gonadotropins, both in pre-pubertal and pubertal fish [9]. In line with these results, KISS1–10 or KISS2–10 treatment with slow release implants produced an upregulation of pituitary expression of *fshβ* and *lhβ* and stimulated gonadal development in yellowtail kingfish (*Seriola lalandi*) pre-pubertal males [39], both within and outside the breeding season. Other positive example of exogenous kisspeptin administration was seen in the chub mackerel (*Scomber japonicus*) after treatment with KISS1 through subcutaneous injections [40]. In this case, gonadosomatic index (GSI), spermatozoa concentration and plasma sex steroids levels significantly increased in treated fish. Although the effects of kisspeptin treatment depend on the gonad stage and the mode of administration [7,9,41,42], altogether the reported results highlight its potential use to induce maturation in fish species with reproductive problems, representing a valuable tool to optimize hormonal induction and breeding protocols. 

In vertebrates, kisspeptin seems to act upstream of HPG axis (reviewed in Beato*,* et al. [43], inducing the release of GnRH at the hypothalamus and stimulating the synthesis and secretion of Lh and Fsh in the anterior pituitary. Lh and Fsh released to the circulatory system reach the gonads and regulate steroidogenesis in the Leydig and theca cells and support gametogenesis in Sertoli and granulosa cells, respectively. In vertebrates, there is a complex and tight regulation of the HPG axis, with several negative feedback regulations from gonads, as well as a tight control of the KISS signaling through neurotransmitters and neuropeptides (reviewed in Beato*,* et al. [43]). Although kisspeptin was found to be essential for mammalian reproduction as a stimulator of hypothalamic GnRH and a regulator of puberty onset [44], it seems that in fish, kisspeptin actions appear not to be mediated by GnRH neurons as in mammals (reviewed in Somoza*,* et al. [8]). Despite of all these positive results of kisspeptin injection on fish HPG axis and gonadal maturation, the underlying mechanism remain to be elucidated. Moreover, since fish with *kiss* and/or *kissr* mutated genes are able to reproduce relatively normally [45,46,47], kisspeptin might act as an enhancer of gonadotropin synthesis and release during fish reproduction through a mechanism that remains to be discovered. Furthermore, although the lack of kisspeptin receptors in GnRH neurons in some teleost species suggests there is no direct neuronal action [47], KISS1 increased spike frequency and depolarized membrane potential of hypophysiotropic GnRH3 neurons, while KISS2 suppressed it, in the brain of adult zebrafish [48].

In the present study, the circulating miRNAs let-7e and miR-199a-3p were up-regulated in Senegalese sole females treated with kisspeptin in comparison with control females, while miR-100-5p was down-regulated. The let-7 family was found to be highly expressed in the gonads of olive flounder (*Paralichthys olivaceus*) [49], pompano (*Trachinotus ovatus*) [50], and roundworm (*Caenorhabditis elegans*) [51], suggesting a functional conservation of its crucial role in reproductive physiology [52]. Let-7e from blood plasma was also found to be up-regulated in Senegalese sole breeders with improved sperm quality [20]. miR-199a-3p has also been correlated with reproduction. While miR-199a, was down-regulated in mature testis of rainbow trout (*Oncorhynchus mykiss*), miR-199a-5p was particularly up-regulated in the brain of mature fish [53]. In contrast to previous reports, where miR-100-5p was more abundant during later stages of gonadal development in ovaries and testis of zebrafish [54], and shown to have putative functions of promoting cell differentiation [55]; we found that it was down-regulated in the plasma of kisspeptin-treated females. Nevertheless, previous studies also link the expression of this miRNA with reproduction in oriental river prawn (*Macrobrachium nipponense*) [56] and the pompano [50]. Indeed, this miRNA was also observed to be involved in oocyte maturation [57], and its over-expression has been associated with the inhibition of T release in mammals [58]. These last results were in concordance with the present study where the application of kisspeptin treatment increased the T plasma levels at 2 d post-injection in females and down-regulated the circulating miR-100. 

Only one circulating mature miRNA (miR-1-3p) was found DE in Senegalese sole males, being more highly expressed in males treated with kisspeptin hormone in comparison with control males. miR-1 has been found to be expressed in both skeletal and cardiac muscle lineages, where its main biological roles are cardiogenesis, myogenesis and skeletal muscle hypertrophy. Humans with cardiac injury have higher circulating levels of this miRNA after acute myocardial infarction [59]. In fish, this miRNA has been reported to show highly conserved tissue-specific expression patterns [60,61] and potentially playing an important role in regulation of muscle gene expression in fish species [60]. 

Only 4 mRNAs potentially targeted by miR-1-3p were identified (*dnmt3ab*, *sptlc1*, *pqlc3*, and *tpm4a*). DNA methylation is crucial for normal development and cellular differentiation in many large-genome eukaryotes [62]. In mammals, both Dnmt3a and Dnmt3b are primarily responsible for the *de novo* DNA methylation. Dnmt3 morpholino-injected zebrafish embryos exhibited small brains, defective pharyngeal arch formation, and abnormal retinal neural epithelial differentiation [63]. Interestingly, although *dnmt3b* transcript levels were higher in metamorphosed Senegalese sole specimens reared at 15 °C than at either 18 or 21 °C, *dnmt3a* paralogue had a uniform expression profile across temperatures [64]. Nevertheless, the impact of thermal regime (comparing 16 °C and 20 °C) on the expression of *dnmt3aa* and *dnmt3ab* was reported in Senegalese sole lecithotrophic larval stages, suggesting they might be involved in thermal programming [65]. *Sptlc1* gene mutations cause a neuropathy known as hereditary sensory neuropathy type I [66], which might be related with its reported role in the sphingolipid metabolism pathway [67]. Sphingolipid metabolism was also previously associated with the Senegalese sole sperm quality improvement through dietary vitamin K supplementation by the analysis of DE sncRNAs in circulation [20]. While nothing is known about the potential role of Pqlc3, Tpm4 has been reported to be involved in the fine tuning of the cellular contraction, and an association with the development of cardiac hypertrophy was suggested [68], in line with the cardiac injury observed in humans with higher circulating levels of miR-1 after acute myocardial infarction [59].

Among the potentially targeted mRNAs by DE miRNAs in females, cell junction protein class, and plasma membrane and cell periphery GO-Slim cellular components were significantly overrepresented. Genes coding for gap junction proteins (or connexins) such as *connexin 32.3* (*cx32.3*) and *connexin 28.9* (*cx28.9*), and tight junction proteins such as *tight junction protein ZO-3* (*tjp3*) and *occludin a* (*oclna*) were found. Connexins form channels between the cells and facilitate the cellular cross talk, connecting the cytoplasm of adjacent cells together allowing ions, small molecules (<1 kDa) and secondary metabolites to be diffused [69]. In mammals, several connexins are known to play an essential role in female reproductive health [70,71]. Although little is known regarding the potential role of connexins in fish reproduction, their role on controlling meiotic arrest of oocytes have been evidenced [72]. In addition to connexins, tight junctions (TJ) are also protein structures that control paracellular permeability to various molecules, including water, ions, and macromolecules, across cell layers; and play a key role in ovarian follicle development (reviewed in Zhang*,* et al. [73]), among other biological processes. Indeed, abundance of *ocln*, *claudins* (*cldns*), and *tjp1* mRNAs changed during follicular growth and are hormonally regulated. Recent reports further suggest that hormonal down-regulation of TJ proteins during ovarian follicular development could reduce barrier function (i.e., allowing the paracellular passage of water and molecules) and allow the increase of follicular fluid volume as well as serum factors going into the follicle [73]. Nevertheless, since the expression domains of genes encoding TJ proteins are highly diverse, including different normal but also neoplastic tissues [74], it is difficult to address whether this bioinformatically prediction is specifically linked to TJ proteins from the female gonadal (granulosa and theca) cells. Besides genes encoding TJ proteins, *slc6a22* solute carrier was also found to be predicted as targets of DE ncRNAs. Slc6 transporters include the serotonin, dopamine, norepinephrine, taurine, creatine, as well as amino acid transporters [75]. While serotonin can control oocyte maturation [76], dopamine is a catecholamine implicated in many functions, mediating sexual motivation in mice [77] and gonadotropin inhibition in fish [78]. Furthermore, it has been recently hypothesized that dopamine might be also involved in the reproductive dysfunction of Senegalese sole [79]. 

In addition to all these proteins regulating cell to cell communication, several genes (including *snx1* and *snx27* as well as *dopey2* and *track1*) encoding intracellular trafficking proteins were also predicted to be targeted by DE miRNAs. Sorting nexins (SNXs) is a growing family of proteins involved in vesicular trafficking between cellular compartments [80]. Furthermore, while Dopey2 has been shown to be involved on normal neural development and functioning [81], Trak1 is involved in the regulation of endosome-to-lysosome trafficking, essential for mitochondria motility [82]. In neurons, the efficient and regulated transport of mitochondria along axons to synapses is crucial for maintaining function. Indeed, gene silencing by targeted shRNAi and dominant negative approaches resulted in impairing mitochondrial mobility in axonal processes [83]. 

Altogether, the *in silico* prediction of mRNAs targeted by DE miRNAs suggests that the kisspeptin treatment of Senegalese sole females might affect paracellular transporters, regulate structural and functional polarity of cells, neural networks and intracellular trafficking. In males, kisspeptin therapy might induce an altered profile of DNA methylation and sphingolipid metabolism. Therefore, the analysis of miRNAs from blood plasma revealed kisspeptin may affect the HPG axis in fish through previously unexpected molecular pathways.

## 4. Materials and Methods 

Experimental procedures were conducted in accordance with the guidelines of the European Directive (2010/63/EU) and Portuguese legislation for the use of laboratory animals, and also considered the ARRIVE guidelines. CCMAR facilities and their staff are certified to house and conduct experiments with live animals (Group-C licenses by Direção Geral de Alimentação e Veterinária—DGAV). The authorization for experimental procedures with germ cells were previously approved by DGAV (ref.0421/000/000/2013). 

### 4.1. Animals and Housing

All experiments took place at the CCMAR Research Station “Ramalhete” (Faro, Portugal), using an established Senegalese sole G1 broodstock, previously sexed according to Cabrita*,* et al. [16] and acclimated to captivity conditions for 2–3 years. Fish were kept indoors in four circular 3 m^3^ tanks, with 1500 L of seawater. Tanks were supplied with flow-through gravel-filtered seawater at a constant flow (±4 L/min). A total of 62 adult *Solea senegalensis* (15–16 individuals per tank) were used, with an average weight of 1311.94 ± 434.03 g, individually identified with a PIT-tag system (ID100 Implantable Transponder, Trovan, Dorset, Aalten, The Netherlands) and maintained at a sex ratio of 1:1. Physical and chemical parameters such as dissolved oxygen saturation (89.7 ± 2.8%), temperature (17.7 ± 2.3 °C) and salinity (34.7 ± 0.7‰) were measured on a daily basis (April–May) to monitor water quality conditions. Natural photoperiod was simulated with a clock system according to environmental conditions (sunrise and sunset respectively set for 6:38 and 20:19 h during samplings) in the area (37°00′23′′ N; 7°58′03′′ W, Faro, Portugal), while temperature naturally oscillated during the two months of experimentation. Individuals were fed 6 out of 7 days of the week during the morning, on commercial artificial pellets (BroodFeed, SPAROS Lda., Olhão, Portugal) at a daily ration of 2% (*w*/*w*) biomass. 

### 4.2. Experimental Design 

To determine the effectiveness of an *in vivo* treatment with kisspeptin as a stimulator of the reproductive axis in Senegalese sole, the present trial was performed in spring (April–May) coinciding with Senegalese sole main reproductive season. According to the previously deduced amino acid sequence of the core kisspeptin-10 region in Senegalese sole proteins, Ss_KISS2_v1 (NH2-FNFNPFGLRF-CONH2, GeneBank HM116743) [25] amidated decapeptide was synthesized by CPC Scientific Inc. (San José, California, USA), with a purity of 95%. Before the administration of the kisspeptin treatment, a first sampling (T0) was performed in all tanks to determine the basal values of the sex hormones studied. In this first sampling, fish were primarily anaesthetized in seawater containing 300 ppm of 2-phenoxyethanol (77699 Fluka, Sigma-Aldrich, St. Louis, MI, USA). When unresponsive to touch, approximately 1 mL of blood was extracted by caudal puncture using heparinized syringes. Plasma was later separated by centrifugation (3000× *g*, 15 min, 4 °C) and frozen at −80 °C until further analysis. In females, the gonadal development stage was scored according to external abdominal swelling, as described previously [84]. In males, a sperm sample was collected as described by Cabrita*,* et al. [16]. In brief, the urogenital pore was dried and sperm was collected with a syringe or with a 20 μL micropipette by gently pressing the testes on the fish blind side. Samples were stored on ice in a styrofoam support until further analysis. Samples contaminated with urine were discarded.

Treatment with kisspeptin was performed 3 days after first sampling. The fish maintained in two of the four tanks (*n* = 31) were intramuscularly injected with KISS2 decapeptide at a dose of 250 µg/kg body weight, based on previous reports of positive results of KISS2-10 eliciting gonadotropin release [9] and gonadal development [38] in fish. The remaining sole were injected with phosphate buffered saline (PBS) to test the placebo effect (control group). The same day, blood samples were collected at 2 and 4 h after treatment to determine the acute effect on hormonal levels. Further samplings were performed at 2 and 4 days after the treatment to collect both blood and sperm samples and to determine the females’ gonadal maturation stages. At this stage, plasma was also collected for detection of circulating microRNAs as previously described in [20]. No visible sign of hemolysis was noted in collected plasma samples. A total of 200 μL of plasma was sampled to perform hormones quantification and stored at −20 °C until further analysis. The remaining plasma was re-centrifuged at 3000× *g* for 5 min (to avoid cell debris contamination) and 500 μL of supernatant plasma was collected, snap-frozen in liquid nitrogen, and stored at −80 °C until RNA isolation and analysis.

To avoid excessive manipulation of fish in a short period of time, one tank from each treatment (*n* = 15/16 fish) were used for samplings at 2 h and 2 d after treatment, while the other tanks were used at 4 h and 4 d.

### 4.3. Sperm Quality Analysis

Sperm quality analysis was performed according to protocols previously optimized in our laboratory for motility and viability [19]. Total motility was determined in all samples using computer assisted sperm analysis (CASA) and ISAS software (ISAS, Proiser R+D, S.L., Valencia, Spain). Motility analysis was performed activating 1 μL of sperm with 10 μL of seawater (21 °C and 35 ppt salinity) in a Makler chamber using a phase-contrast microscope (Nikon 200, Tokyo, Japan) with a 10× negative contrast objective and a digital camera (Basler A312f C-mount, Ahrensburg, Germany) set for 50 fps. The settings for CASA software were previously adapted for this species. CASA parameters registered were percentage of motile cells (TM; %) and velocity according to the actual path (VCL; μm/s). Motility parameters were assessed at 15, 30, 45, and 60 s post-activation.

To assess sperm viability, 2 µL of sperm were diluted in 500 µL of 1% NaCl in flow cytometer tubes and propidium iodide (PI-Sigma, City, Spain) was added at 1 μg/mL final concentration to detect dead cells. Immediately after, samples were acquired in a flow cytometer (FACSCalibur, BD Biosciences, CA, USA) adjusted for blue excitation (488 nm) line for the detection of PI (670/30). Flow cytometer settings and gates were previously adjusted using a positive (100% dead cells) and a negative control. Data analysis was performed applying Weasel 3.1 free software. A total of 75,000 events were counted for each sample. The percentage of viable cells was recorded. At least 6 samples from individual males were analyzed at each sampling point.

### 4.4. Analysis of Hormones in Blood Plasma

The levels of gonadotropins (Fsh and Lh) and sex steroids (E_2_ and T in females; 11-KT and T in males) in plasma samples were determined by enzyme-linked immunosorbent assay (ELISA), according to previously optimized protocols for this species [15,16,20]. 

Plasma levels of endogenous gonadotropins were determined in duplicates by competitive ELISAs, following previously described protocol using recombinant Senegalese sole Fsh and Lh (rFsh and rLh, respectively) and specific antibodies against sole Fshβ and Lhβ subunits [15,85]. Circulating levels of 11-KT, T, and E_2_ were assessed by the respective ELISA kits from Cayman Chemicals (Ann Arbor, Michigan, United Stated), according to the manufacturer’s protocol from duplicate plasma samples and as described elsewhere [20]. For each hormone measured a total of 6–9 samples were analyzed per treatment, sex, and time point.

### 4.5. microRNAs Analysis

#### 4.5.1. Isolation, Libraries Preparation, and Sequencing

Small RNA sequencing (sRNA-Seq) was performed on RNA isolated from blood plasma from Senegalese sole treated (*n* = 16) and non-treated with kisspeptin (*n* = 15) (see details in Table 3). SncRNAs were isolated from blood plasma samples using miRNeasy Serum/Plasma Kit (Qiagen, Germany) following the manufacturer’s instructions, and assessment of RNA quality and quantity was performed on a 2200 TapeStation Nucleic Acid system using High Sensitivity RNA ScreenTapes (Agilent, Santa Clara, CA, USA). Libraries were prepared from 30 samples (Table 3) using NEXTflex Small RNA-Seq kit v3 (Bio Scientific, Phoenix, USA) for Illumina platforms following the manufacturer’s protocol. Library size, purity, and concentration were evaluated on a High Sensitivity D1000 ScreenTape (Agilent, USA). Normalized libraries were pooled at equimolar concentrations and multiplexed sequencing was done in two independent runs on a NextSeq500 sequencer using a NextSeq High Output kit v2 (75 cycles; Illumina, San Diego, CA, USA). All sequencing data were submitted to the NCBI SRA database under the accession number GSE153469. 

#### 4.5.2. Data Processing and Annotations

Sequencing data were first trimmed with Cutadapt version 2.8 [86] using the parameters -a TGGAATTCTCGGGTGCCAAGG to remove the adapters and -u 4 and -u -4 for removal of random bases from the beginning or end of each read, respectively. The trimmed reads were mapped to the hairpin sequences of miRbase (version 21, http://www.mirbase.org/) using bowtie2 with parameter -k10 (PMID: 22388286 [87]). The mapped reads were further annotated using the gff files for mature miRNAs in miRbase to identify mature miRNAs from multiple species (ftp://ftp.ebi.ac.uk/pub/databases/RNAcentral/current_release/genome_coordinates/gff3/). In those cases where the same miRNA-ID was identified in multiple species, the miRNA was chosen from the species with highest expression of that miRNA as the correct miRNA. This left us with an expression matrix of uniquely expressed miRNAs. Other ncRNAs than miRNAs were identified using the RNACentral database (https://rnacentral.org/). First the reads were mapped to the tongue sole and zebrafish genome and then the zebrafish gff in RNACentral was used to identify ncRNAs. DE RNAs were identified using Limma-voom in R (https://genomebiology.biomedcentral.com/articles/10.1186/gb-2014-15-2-r29). The analysis required the miRNA to be expressed with at least 1 count per million (cpm) in all samples. The Trimmed Mean of M-values (TMM) normalization was used when calculating the normalization-factors. *p*-values were adjusted using Benjamin–Hochberg correction and significance was set at 0.05.

#### 4.5.3. mRNA Target Prediction

To predict mRNAs targeted by DE miRNAs in Senegalese sole males and females when treated or not with kisspeptin, an assembled transcriptome of *S. senegalensis* (http://www.juntadeandalucia.es/agriculturaypesca/ifapa/soleadbifapa/) was used. To explore potential mRNAs targets, the corresponding 5′ and 3′ UTR regions and the coding sequence (CDS), were considered. Potential mRNA binding sites for miRNAs were identified using RNAhybrid [88]. An energy threshold of ≤−26 kcal mol^−1^ and a strict seed matching (no G:U allowed) in 2–8 nt from the miRNA 5′ end were applied. Seeding regions (2–8 nt) lengths were considered based on previous studies on miRNAs [89,90]. Gene ontology (GO), overrepresentation (Fisher’s exact test with Bonferroni correction for multiple testing; *p* < 0.05), and pathway analysis of predicted mRNAs were done using the Panther (http://www.pantherdb.org/) and Kyoto Encyclopedia of Genes and Genomes (KEGG; http://www.genome.jp/kegg/) platforms.

### 4.6. Statistical Analysis

Statistical analysis and data plotting were performed using Microsoft Excel and SPSS. Hormone levels were expressed as means ± standard error of the means (SEM). All these data sets were tested for normal distribution using the Shapiro-Wilk test [91]. The concentration of each hormone along the different sampling points within each group were then tested for significant differences using a one-way ANOVA, followed by a Dunnett’ test performing a pair-match multiple comparison procedure comparing the time points 2 d and 4 d with T0 as a control point. A Student’s *t-*test was also applied for comparisons between groups (kisspeptin treated and control) within each sampling point. To compare sperm motility parameters among groups, a general linear model with Bonferroni correction was used. In all cases statistical significance was set at *p* < 0.05.

## 5. Conclusions

In the present study, kisspeptin treatment stimulated gonadotropin synthesis and secretion, as well as the testosterone plasma levels, in both Senegalese sole genders. However, further optimization of the protocol is required to solve Senegalese sole G1 male dysfunction and fully trigger the courtship behavior observed in wild breeders. Kisspeptin treatment also induced an alteration of several miRNAs in plasma. While in kisspeptin treated females let-7e and miR-199a-3p were up-regulated and miR-100-5p down-regulated, in males the expression of miR-1-3p was up-regulated. Bioinformatic prediction of mRNAs targeted by these sncRNAs opened new research avenues to understand how kisspeptin might impact HPG axis in fish species, and hypothetically overcome G1 males reproductive dysfunction. The kisspeptin treatment appears to be a very promising therapy to induce Senegalese sole reproduction but more research is still needed, namely testing repeated treatments or slow-release implants.

## Figures and Tables

**Table 1:** Cell viability of sperm samples from Senegalese sole males treated with kisspeptin (*n* = 17) and control (untreated) (*n* = 13), before (T0) and after (2 d: 2-days; 4 d: 4-days) the kisspeptin injection.

Sampling	Treatment	Live Cells (%)
T0	Kiss	88.55 ± 12.77
	Control	90.47 ± 4.81
2 d	Kiss	84.87 ± 6.40
	Control	89.32 ± 2.96
4 d	Kiss	89.75 ± 4.26
	Control	88.53 ± 1.35

**Table 2:** Differentially expressed isomiRs between kisspeptin and control in Senegalese sole females. Canonical mature miRNA names (from miRBase 21.0) are indicated, along with the sequence of the differentially expressed miRNAs, their log2 fold change of the kisspeptin–control statistical comparison (corresponding to the log2 of the miRNAs’ average expression in Kisspeptin subtracting the average expression in control values computed by limma) and the isomiRs average log2 cpm in the dataset, as computed by limma. Benjamin–Hochberg adjusted *p*-values are also indicated.

miRNA	Sequence	Log2 Fold Change	Average Expression	Adjusted *p*-Value
let-7e	TGAGGTAGTTGGTTGT	6.295	2.352	0.00939
miR-199a-3p	AGTAGTCTGCACATTGGTT	7.973	3.232	0.00165
miR-100-5p	AACCCGTAGATCCGAACTTGTG	−9.335	4.472	0.00006

**Table 3:** Canonical summary of the sequenced circulating miRNAs in Senegalese sole females and males treated with kisspeptin hormone and control (untreated) during the different sampling points (2 d: 2-days and 4 d: 4-days post-injection). The raw reads, data after trimming (trimmed, trimmed data in percentage) and annotated microRNA reads and microRNA data in percentage are also indicated.

**Kisspeptin** **treated**	**Sample ID**	**Sex**	**Sampling** **Point**	**Raw Reads**	**Trimmed**	**Trimmed %**	**microRNA**	**microRNA (%)**
	A1-1_S1	Female	2 d	453,229	208,921	46	47,725	11
	A1-2_S2	Female	2 d	500,647	237,779	47	91,581	18
	A1-3_S3	Female	2 d	567,594	288,477	51	129,980	23
	A1-4_S4	Female	2 d	475,441	209,746	44	57,941	12
	A1-11-9_S5	Female	2 d	448,350	219,818	49	25,468	6
	A1-6_S11	Male	2 d	409,534	199,896	49	57,262	14
	A1-7_S12	Male	2 d	438,943	207,974	47	57,138	13
	A1-10_S13	Male	2 d	497,364	252,675	51	63,137	13
	A1-12_S14	Male	2 d	500,206	222,774	45	48,918	10
	A1-13_S15	Male	2 d	431,890	200,621	46	35,429	8
	A1-11-12_S16	Male	2 d	448,502	218,492	49	157,656	35
	A4-3_S26	Male	4 d	509,277	258,979	51	139,680	27
	A4-8_S27	Male	4 d	395,813	180,599	46	41,197	10
	A4-9_S28	Male	4 d	505,458	256,239	51	104,044	21
	A4-10_S29	Male	4 d	498,680	233,040	47	49,651	10
	A4-17_S30	Male	4 d	463,867	223,033	48	85,875	19
**Control** **untreated**	A2-2_S6	Female	2 d	461,160	227,736	49	191,846	42
	A2-5_S7	Female	2 d	491,359	243,485	50	163,635	33
	A2-7_S8	Female	2 d	415,844	192,516	46	124,752	30
	A2-9_S9	Female	2 d	475,556	211,070	44	84,252	18
	A2-10_S10	Female	2 d	443,098	218,571	49	52,960	12
	A2-11_S17	Male	2 d	496,908	245,995	50	39,267	8
	A2-12_S18	Male	2 d	488801	233,186	48	65,358	13
	A2-14_S19	Male	2 d	549,711	227,754	41	29,918	5
	A2-16_S20	Male	2 d	502,982	226,236	45	41,948	8
	A3-2_S21	Male	4 d	445,074	219,334	49	47,514	11
	A3-5_S22	Male	4 d	500,306	248,426	50	93,047	19
	A3-6_S23	Male	4 d	464,452	219,739	47	98,906	21
	A3-9_S24	Male	4 d	470,313	232,473	49	133,423	28
	A3-11_S25	Male	4 d	488,090	247,258	51	31,354	6

## Supplementary Materials

Supplementary Materials can be found at https://www.mdpi.com/1422-0067/21/23/9051/s1, Figure S1: Overview of sequencing results. (**A**) Detected fragment lengths in the finished sequencing libraries for all samples. The intensities of each length are colored by rpm (reads per million) for which darker color indicates higher expression of that particular fragment length. (**B**) Distribution of detected RNA types in the different samples. lncRNA: long non-coding RNA; rRNA: ribosomal RNA; SRP_RNA: signal recognition particle RNA; miRNA: micro RNA; snRNA: small nuclear RNA; tRNA: transfer RNA; Table S1: Senegalese sole mRNA targeted by DE miRNAs. The annotated Sengalese sole unigene and gene name are shown; Table S2: Pathways represented by Senegalese sole mRNAs targeted by DE miRNAs. The different pathways, number of genes implicated and the percentage are indicated.
